# Podophyllotoxin Alleviates DSS-Induced Ulcerative Colitis *via* PI3K/AKT Pathway Activation

**DOI:** 10.33549/physiolres.935487

**Published:** 2025-04-01

**Authors:** Ting LI, Xiaoying WANG, Jiawei WANG

**Affiliations:** 1 Department of Emergency, The Affiliated Hospital of Southwest Medical University. Luzhou, Sichuan, China

**Keywords:** Podophyllotoxin, Ulcerative Colitis, Inflammation, PI3K/AKT

## Abstract

This study systematically evaluated the therapeutic effects of podophyllotoxin in a DSS-induced mouse model of ulcerative colitis. A total of 374 podophyllotoxin-related targets were identified through database screening, and by intersecting them with 1,741 UC-related targets, 120 potential therapeutic targets were obtained. Subsequent GO and KEGG enrichment analyses revealed that these targets are primarily involved in biological processes such as the positive regulation of protein kinase B signaling, cellular response to lipopolysaccharide, and inflammatory responses, with significant enrichment in key pathways like the PI3K-Akt signaling pathway. Molecular docking results indicated that podophyllotoxin has strong binding activity with several targets related to inflammation and signal transduction. Animal experiments further validated the significant therapeutic effects of podophyllotoxin in the DSS-induced ulcerative colitis mouse model. Particularly at high doses, podophyllotoxin effectively alleviated ulcerative colitis symptoms, reduced pathological damage to colonic tissues, and enhanced intestinal barrier function. Additionally, podophyllotoxin significantly lowered the levels of inflammatory cytokines (TNF-α, IL-1β, IL-6) in the serum and colonic tissues of ulcerative colitis model mice and improved oxidative stress status. More importantly, podophyllotoxin effectively restored the impaired intestinal mucosal barrier function by enhancing the expression of tight junction proteins such as ZO-1 and occludin. Finally, the study revealed that podophyllotoxin may alleviate ulcerative colitis symptoms and promote colonic tissue repair by activating the PI3K/AKT signaling pathway. These findings provide strong experimental evidence for the potential use of podophyllotoxin as a therapeutic agent for ulcerative colitis and offer valuable insights for the future development of ulcerative colitis treatment strategies targeting the PI3K/AKT pathway.

## Introduction

Ulcerative Colitis (UC) is a chronic inflammatory bowel disease primarily affecting the mucosal layer of the colon and rectum, characterized by persistent inflammation of the colonic mucosa, usually starting from the rectum and extending proximally [1, 2]. The clinical manifestations of UC are diverse, including diarrhea, abdominal pain, rectal bleeding, tenesmus, and systemic symptoms such as fever, weight loss, and anemia [3, 4]. The incidence of UC is relatively high worldwide, particularly in North America and Europe, with an annual incidence rate of approximately 8–14 per 100,000 individuals [5]. Although current treatments can alleviate UC symptoms to some extent, various drugs and surgical options have limitations and side effects [6]. In terms of pharmacological treatment, long-term use of 5-ASA, corticosteroids, and immuno-suppressants may lead to severe side effects, increasing the risk of infections and liver and kidney damage [7, 8]. While biologics are highly effective, their widespread use is limited by high costs and potential serious side effects. Surgical intervention, although curative for UC, may result in a decline in quality of life and long-term complications post-surgery [9, 10]. Given the limitations and side effects of existing therapies, researching and developing new treatments, particularly natural medicines with anti-inflammatory and mucosal protective effects, is of great significance for improving the quality of life of UC patients.

Podophyllotoxin (PI) is a lignan compound extracted from plants [11]. The structure of PI contains multiple hydroxyl and methoxy groups, which confer it with certain polarity and biological activity [12]. Modern pharmacological research has demonstrated that PI possesses a wide range of biological activities, particularly in the fields of antitumor and antiviral applications [12–14]. PI exerts its antitumor effects by inhibiting tubulin polymerization, thereby preventing cell mitosis and causing cell cycle arrest at the G2/M phase [15, 16]. Additionally, Etoposide and teniposide, derivatives of podophyllotoxin, have been approved by the FDA for the treatment of a wide range of cancers, including lung cancer, testicular cancer, and lymphoma [11, 13]. The anti-inflammatory effects of PI are primarily mediated by inhibiting the production and release of pro-inflammatory cytokines, regulating the balance of inflammatory mediators, and reducing tissue inflammatory responses [17]. Some in vitro and in vivo studies have shown that PI can inhibit the expression of pro-inflammatory factors such as TNF-α, interleukin-6 (IL-6), and IL-1[17]. Furthermore, PI can reduce the release of inflammatory mediators by inhibiting the NF-κB and MAPK signaling pathways [18, 19]. Its anti-inflammatory, antioxidant, and immunomodulatory effects make it a potential candidate for the treatment of inflammatory bowel diseases (IBD) such as UC.

**Fig. 1:**
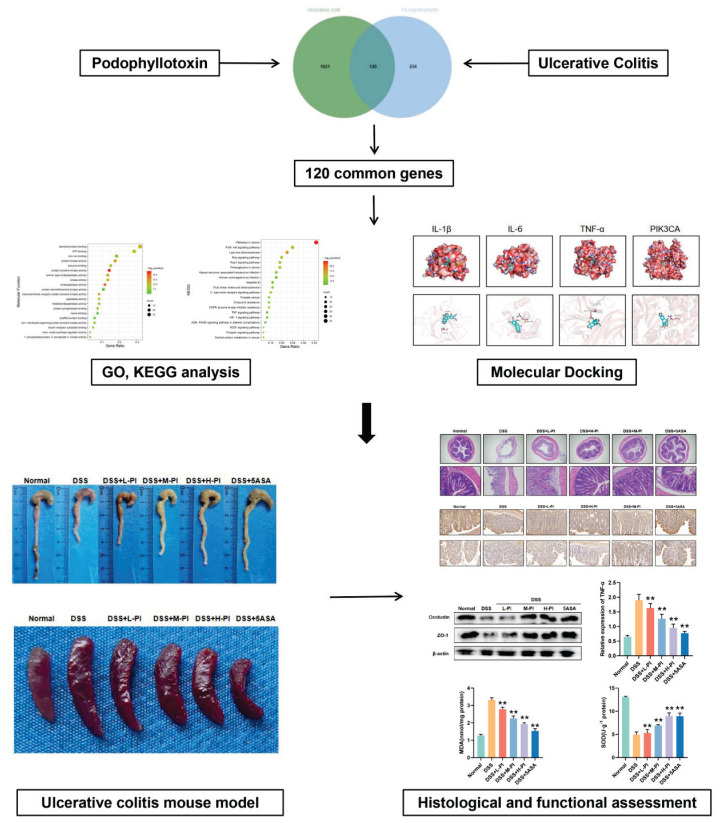
The detailed flow chart of the study.

This study aims to identify and validate potential molecular targets of podophyllotoxin (PI) in the treatment of ulcerative colitis (UC) for the first time, with a focus on its anti-inflammatory effects and clinical application potential using an animal model. Additionally, this study will investigate the specific mechanisms underlying action of PI in UC, particularly its regulatory effects on inflammatory signaling pathways. The innovation of this study lies in addressing the existing research gaps by identifying molecular targets of PI, thereby providing new natural therapeutic options for UC treatment. This will expand current treatment strategies and offer new perspectives and approaches for UC management. A schematic of the workflow is illustrated (Fig. 1).

## Methods

### Prediction of PI and UC-related targets

First, the molecular structure of PI is obtained from PubChem (https://pubchem.ncbi.nlm.nih.gov/), and then the molecular structure is imported into the Swiss Target Prediction database (http://www.swisstargetprediction.ch) to collect the corresponding targets of PI. Using “ulcerative colitis” as the keyword, search for UC-related targets in the GeneCards database (http://www.genecards.org), DisGeNET database (https://www.disgenet.org), and OMIM database (http://omim.org). From the GeneCards search results, targets with a relevance score ≥5 are selected and merged with the search results from DisGeNET and OMIM databases. After removing duplicates, the targets related to ulcerative colitis are obtained. The intersection of PI targets and UC-related targets is then determined using the online tool Venny 2.1 (https://bioinfogp.cnb.csic.es/tools/venny/).

### GO and KEGG Enrichment Analysis

The intersection targets are subjected to GO and KEGG enrichment analysis using the DAVID database (https://david.ncifcrf.gov/). GO analysis is employed to explore the biological functions of the gene targets, while KEGG pathway analysis identifies potential relevant pathways, elucidating the molecular mechanisms of PI in treating ulcerative colitis from a molecular biology perspective. The results of GO and KEGG analyses are visualized using the online tool (http://www.bioinformatics.com.cn). The data on PI, ulcerative colitis, KEGG pathways, and the involved intersection targets are then integrated and imported into Cytoscape 3.9.1 software to construct a “Drug-Target-Disease-Pathway” network diagram.

### Molecular docking

The three-dimensional structures of the core targets TNF-α (6OP0), IL-6 (4O9H), IL-1β (1TWM), AKT1 (6hhg), AKT2 (3E87), PIK3CA (5XGI), and PIK3CG (2A5U) are obtained from the PDB database (https://www.rcsb.org/). Using Pymol 2.5.1 software, small molecules are removed from the structures. The proteins are then prepared in AutoDock 4.2.6 by removing water molecules, adding hydrogens, and saving the structures as PDBQT protein receptor files. The three-dimensional structure of PI is retrieved from the PubChem online database (https://pubchem.ncbi.nlm.nih.gov/) and processed similarly in AutoDock 4.2.6. The processed proteins and small molecules are then docked using Autodocking, with Auto Dock Vina 1.1.2 employed to evaluate the binding affinity between PI and the targets. A lower score indicates a higher likelihood of binding. Finally, the docking results are visualized and analyzed using Pymol 2.5.1.

### Material

PI was purchased from MCE. Dextran sulfate sodium (DSS) (molecular weight: 40,000 Da) was obtained from Macklin Inc.; 4 kDa FITC-Dextran was purchased from Sigma Aldrich, and Mesalazine (5-ASA) was obtained from Ethy-pharm Pharmaceutical Co Ltd. Hematoxylin and Eosin (H&E) staining reagents, MDA assay kit, SOD assay kit, lysis buffer, BCA protein concentration assay kit, and primary antibody dilution buffer were purchased from Beyotime Biotechnology. FITC-Dextran was also obtained from Sigma. ELISA kits (for TNF-α, IL-1β, IL-6) were purchased from R&D Systems (Lianke Bio). GAPDH, ZO-1, occludin, PI3K, P-PI3K, AKT, and P-AKT antibodies were purchased from Wuhan Sanying Biotechnology. PVDF membranes and ECL reagent were obtained from Millipore.

### Animals and experimental design

Sixty male C57BL/6J mice, aged 8 weeks and weighing 20–22 g, were purchased from Ensiweier Biotechnology Co., Ltd. The mice were housed under conditions of 25±2 °C temperature, 55±5 % relative humidity, and a 12-hour light/dark cycle. All animal studies and experimental procedures were approved by the Animal Ethics Committee of Southwest Medical University (Ethics Approval Number: swmu20240046). The experimental groups were as follows:

Normal Group: Mice were given free access to double-distilled water for 14 days. Model Group (DSS): Mice were given free access to a 2 % DSS solution prepared with double-distilled water for 7 days, followed by 7 days of double-distilled water. Low-Dose PI Treatment Group (DSS+L-PI): Mice were given free access to a 2 % DSS solution for 7 days, followed by double-distilled water. PI was administered via gavage at a dose of 20 mg/kg PI for 7 days. Medium-Dose PI Treatment Group (DSS+M-PI): Mice were given free access to a 2 % DSS solution for 7 days, followed by double-distilled water. PI was administered via gavage at a dose of 40 mg/kg PI for 7 days. High-Dose PI Treatment Group (DSS+H-PI): Mice were given free access to a 2 % DSS solution for 7 days, followed by double-distilled water. PI was administered via gavage at a dose of 60 mg/kg PI for 7 days. Positive Control Group (DSS+5ASA): Mice were given free access to a 2 % DSS solution for 7 days, followed by double-distilled water. On the 7th day, 5ASA was administered via gavage at a dose of 50 mg/kg for 7 days. During the experiment, the body weight, stool consistency, and fecal bleeding of mice in each group were recorded daily, and the DAI score was calculated as follows: DAI score = (weight loss score + stool consistency score + fecal blood score)/3 [20]. At the end of the experiment, the colon and spleen of mice from each group were collected, the colon length was measured, and the spleen index was calculated as the spleen weight (mg) / body weight (g).

### Hematoxylin and eosin (HE) staining

After the modeling phase, the colon tissues of the mice were collected and washed three times with pre-cooled PBS. The tissues were then fixed in 4 % paraformaldehyde, embedded in paraffin, and sectioned into 5 μm thick slices. These paraffin sections were stained using a Hematoxylin-Eosin (HE) staining kit. The stained sections were observed under a microscope, and images were captured.

### FITC-dextran tracing method for measuring intestinal permeability in mice

Before the experiment, the mice were fasted and deprived of water for 4 hours. Each group of mice was then administered 0.5 g/kg FITC-Dextran via gavage. Four hours later, blood was collected via cardiac puncture, and the samples were centrifuged at room temperature to obtain serum. The fluorescence intensity of the serum was measured using a microplate reader at 490 nm and 525 nm.

### Enzyme-Linked Immunosorbent Assay (ELISA)

After the modeling phase, serum was collected from each group of mice. Following the instructions provided in the ELISA kit, the concentrations of IL-6, IL-1β, and TNF-α in the serum were measured using a microplate reader at 450 nm.

### Tissue MDA and SOD Detection

The collected colon tissues were homogenized in lysis buffer, and the supernatant was collected by centrifugation. According to the instructions provided in the assay kits, the samples were prepared and analyzed. The content of MDA was measured using a microplate reader at 532 nm, and the content of SOD was measured at 450 nm.

### Western blot

RIPA lysis buffer, protease inhibitors, and phosphatase inhibitors were prepared in the appropriate ratios and used to lyse colon tissues. The protein concentration of each group was determined using the BCA method. Proteins were separated using SDS-PAGE gels and then transferred onto PVDF membranes. The membranes were blocked with 5 % BSA at room temperature for 90 minutes, followed by incubation with the appropriate primary antibodies overnight at 4 °C. The next day, the primary antibodies were collected, and the membranes were washed three times with TBST. Subsequently, the membranes were incubated with the corresponding secondary antibodies at room temperature for 1 hour, followed by three washes with TBST. Finally, the membranes were developed using a chemilu-minescence imaging system. The primary antibodies used in this experiment included: β-actin (1:3000), ZO-1 (1:1000), occludin (1:1000), PI3K (1:1000), p-PI3K (1:1000), AKT (1:1000), and p-AKT (1:10000).

### Immunohistochemistry

The prepared paraffin-embedded colon tissue sections were deparaffinized, dehydrated, and cleared. Antigen retrieval was performed using sodium citrate buffer, followed by treatment with 0.3 % H_2_O_2_ for 20 minutes. The sections were incubated overnight at 4 °C with primary antibodies ZO-1 (1:200) and occludin (1:200). The next day, the sections were incubated at room temperature for 1 hour with HRP-conjugated goat anti-rabbit IgG secondary antibody (1:3,000). The sections were then stained with 3,3′-diaminobenzidine (DAB) at room temperature for 5 minutes and counterstained with hematoxylin. Finally, the sections were observed under a microscope, and images were captured.

### Statistical analysis

Statistical analysis was performed using GraphPad Prism 9.0. The results have been represented as. Prior to statistical comparison, the normality of the data distribution was assessed using the Shapiro-Wilk test. For data that met the assumptions of normality of variance, differences between two groups were analyzed using the independent samples T-test, while differences among three or more groups were analyzed using one-way ANOVA followed by Tukey’s multiple comparison test. P < 0.05 were considered statistically significant.

## Results

### Screening of potential targets for PI in the treatment of UC

**Fig. 2:**
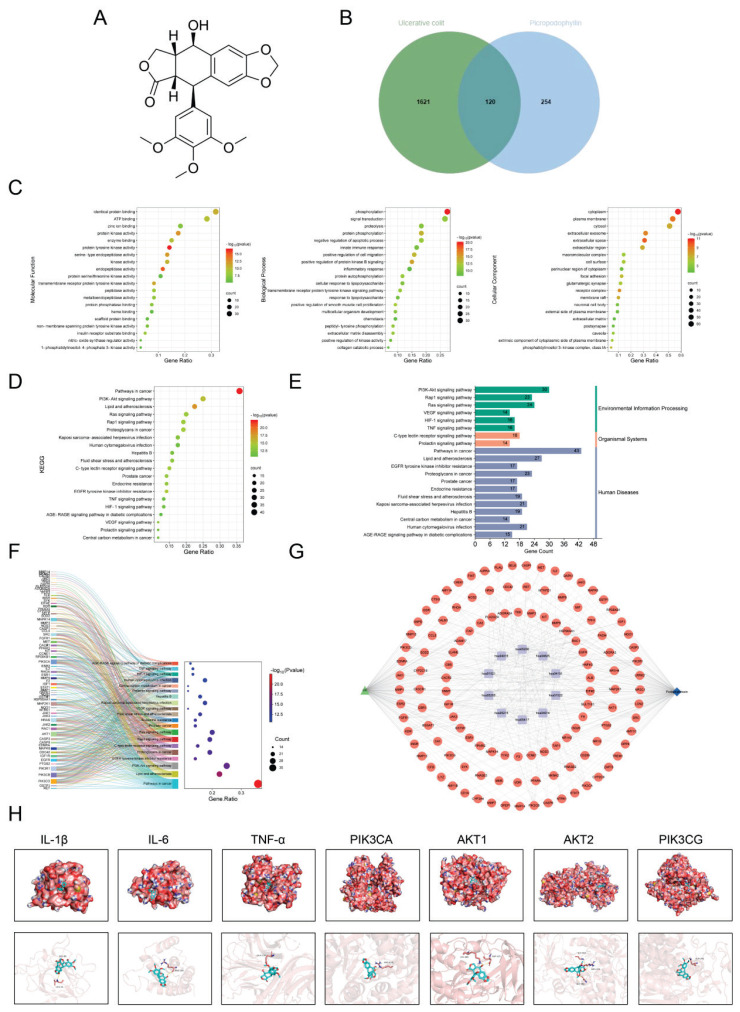
Potential Targets and GO Analysis. **A**) The structure of Podophyllotoxin. **B**) Intersection Targets of Podophyllotoxin and Ulcerative Colitis. **C**) GO Analysis of Intersection Targets. **D–F**) Select the top 20 entries, and create a bubble chart, a classification chart, and a Sankey diagram. **G**) “ Drug - Target - Disease - Pathway “ network diagram. **H**) Visualization of molecular docking.

The molecular formula of PI is shown in Fig. 2A. We obtained 374 targets related to PI from the Swiss Target Prediction database (Table 1). From the GeneCards, DisGeNET, and OMIM databases, 1741 targets related to ulcerative colitis were identified, and duplicates were removed (Table 1). Using the online tool Venny 2.1, the intersection of PI targets and UC-related targets yielded 120 common targets (Fig. 2B). These 120 targets are considered the potential targets of PI for the treatment of UC (Table 1).

### GO analysis and KEGG analysis

We conducted GO enrichment analysis and KEGG enrichment analysis on the 120 intersecting targets using the DAVID database. The GO enrichment analysis identified a total of 618 GO terms (P<0.05), including 438 terms related to biological processes (BP), primarily enriched in positive regulation of protein kinase B signaling, cellular response to lipopolysaccharide, inflammatory response, among others. For cellular components (CC), 69 terms were identified, with significant enrichment in the cytoplasm, phosphatidylinositol 3-kinase complex, class IA, receptor complex, and others. In terms of molecular function (MF), 111 terms were identified, mainly enriched in protein tyrosine kinase activity, endopeptidase activity, and protein kinase activity (Fig. 2C). KEGG pathway enrichment analysis identified 152 significantly related KEGG pathways (P < 0.05). The primary pathways enriched include the PI3K-Akt signaling pathway, Lipid and atherosclerosis, and Pathways in cancer, among others. Sort the items by -log10(p) values, select the top 20 entries, and create a bubble chart (Fig. 2D), a classification chart (Fig. 2E), and a Sankey diagram (Fig. 2F). The data on PI, ulcerative colitis, KEGG pathways, and the involved intersection targets are then integrated and imported into Cytoscape 3.9.1 software to construct a “Drug-Target-Disease-Pathway” network diagram (Fig. 2G).

### Molecular docking

Molecular docking was performed to explore the potential binding targets of PI with the core targets. Typically, a binding energy of less than 0 indicates that there is binding activity between molecules, and a binding energy of less than −5.0 kcal·mol-1 suggests strong binding activity. The results showed that PI exhibited good binding activity with TNF-α (6OP0), IL-6 (4O9H), IL-1β (1TWM), AKT1 (6HHG), AKT2 (3E87), PIK3CA (5XGI), and PIK3CG (2A5U). The binding energies were −6.5 kcal/mol, −7.3 kcal/mol, −7.2 kcal/mol, −7.4 kcal/mol, −7.1 kcal/mol, −8.2 kcal/mol, and −8 kcal/mol, respectively. The docking results were visualized using PyMOL, as shown in Fig. 2H.

### PI Alleviates DSS-Induced UC in Mice

**Fig. 3:**
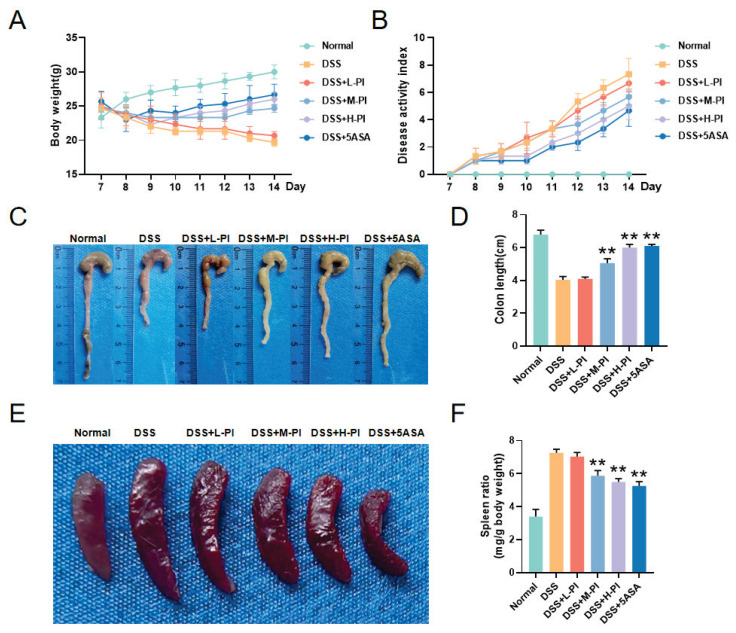
PI Alleviates DSS-induced UC in Mice. **A**) Body weight change curves of mice in each group. **B**) DAI scores of mice in each group. **C–D**) Images of colon length in each group of mice. **E–F**) Images of spleens in each group of mice. Data presented as mean ± SE (n=10). ** P< 0.01, vs the DSS group.

In this study, we investigated the therapeutic effects of PI using a DSS-induced mouse model of UC. The normal group of mice was maintained on a regular diet, and their weight changes were recorded as baseline data. Compared to the normal group, the DSS group exhibited typical UC symptoms, including reduced food intake, continuous weight loss, severe diarrhea, bloody stools, and mucus discharge, confirming the successful establishment of the UC model. Following treatment with PI, a significant dose-dependent protective effect was observed. Specifically, PI markedly slowed the DSS-induced weight loss and improved the physiological condition of the mice (Fig. 3A). Notably, mice treated with a high dose of PI had significantly lower Disease Activity Index (DAI) scores compared to the DSS group, with effects comparable to those of the positive control drug, 5-ASA (Fig. 3B).

Further studies revealed that the colon length in the model group mice was significantly reduced, a typical hallmark of the inflamed state in UC. In contrast, mice treated with high-dose (H-PI), medium-dose (M-PI), or 5-ASA showed significant improvements in colon structure and length. However, mice treated with low-dose PI (L-PI) did not exhibit significant improvements in colon length, and the results were not statistically significant. These findings indicate a significant dose-dependent relationship in the therapeutic effects of the drug (p < 0.01). No significant differences were observed between the high-dose PI group (H-PI) and the 5-ASA group, suggesting that the therapeutic effects of the two treatments are similar (Fig. 3C–D).

Moreover, the development of UC may be accompanied by immune system dysregulation, leading to an increase in spleen size. This was also validated in our experiment [21]. As shown in Fig. 3E–F, the spleen index of the DSS group mice was significantly increased compared to normal mice, indicating the presence of inflammation. However, after treatment with 5-ASA, high-dose, or medium-dose PI, the spleen index was significantly reduced (p < 0.01), demonstrating that these treatments effectively mitigate the inflammatory response (Fig. 3E–F).

### PI Alleviates DSS-Induced Histopathological Damage in the Intestinal Mucosa and Improves Intestinal Barrier Function in UC Mice

**Fig. 4:**
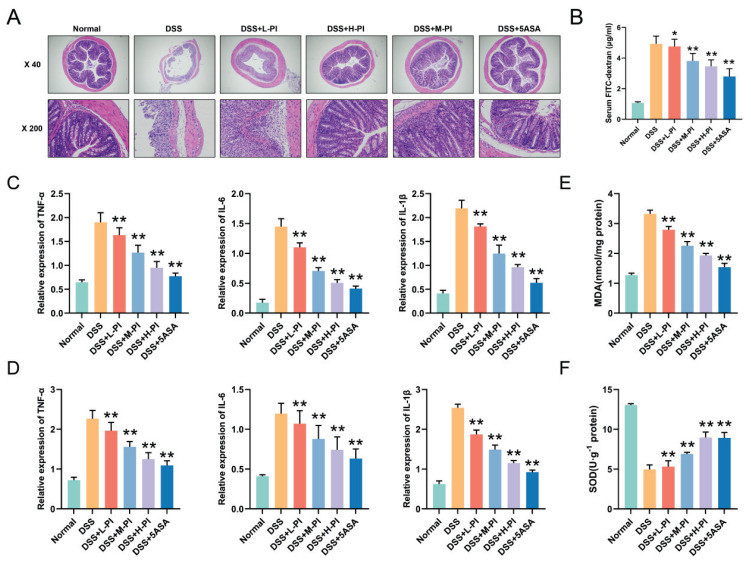
PI alleviates intestinal dysfunction and inflammatory response in UC mice. **A**) HE staining of intestinal tissue in Each group of mice. **B**) Serum FITC-Dextran levels in each group of mice. **C**) Serum levels of TNF-α, IL-1β, and IL-6 in each group of mice. **D**) Levels of TNF-α, IL-1β, and IL-6 in the intestines of each group of mice. **E**) MDA levels in the intestines of each group of mice. **F**) SOD levels in the intestines of each group of mice. Data presented as mean±SE (n=10). ** P< 0.01, vs the DSS group.

To further explore the protective effects of PI on colonic injury in a UC mouse model, histological evaluations were conducted. HE staining results showed that the colonic tissue structure in normal group was healthy, with clearly arranged mucosal epithelial cells and no signs of inflammation. In contrast, the colonic tissue of UC model mice exhibited significant tissue destruction, including distorted glandular structures, crypt necrosis, and extensive infiltration of inflammatory cells. After treatment with PI, the colonic tissue structure was significantly restored, mucosal damage was repaired, glandular structures were reconstructed, and the infiltration of inflammatory cells was markedly reduced. Particularly in the high-dose group, the improvement effects were similar to those observed in the 5-ASA group (Fig. 4A).

To further confirm the potential protective effects of PI on intestinal barrier function, we measured the concentration of FITC-dextran in the serum. The results showed that serum FITC-dextran levels were significantly elevated in the UC model group, indicating impaired intestinal barrier function. However, after treatment with PI, the FITC-dextran levels decreased in a dose-dependent manner, suggesting that PI effectively promoted the repair of the intestinal barrier (Fig. 4B).

### PI reduces inflammatory cytokine secretion and alleviates oxidative stress in DSS-Induced UC mice

To further elucidate the anti-UC effects of PI, we measured the levels of inflammatory cytokines (TNF-α, IL-1β, IL-6) in the serum and colon tissue, as well as oxidative stress markers (MDA and SOD). The results showed that the levels of TNF-α, IL-1β, and IL-6 were significantly elevated in the DSS group, indicating a strong inflammatory response. However, after treatment with PI, the secretion of these pro-inflammatory cytokines decreased in a dose-dependent manner (Fig. 4A-B). Additionally, PI significantly reduced MDA levels (Fig. 4C) in the colon tissue while increasing SOD activity (Fig. 4D), particularly in the high-dose group, where the effects were comparable to those of 5-ASA. These findings suggest that PI has significant potential in mitigating the inflammatory response and oxidative stress associated with UC.

### PI enhances the expression of tight junction proteins in DSS-induced UC mice

**Fig. 5:**
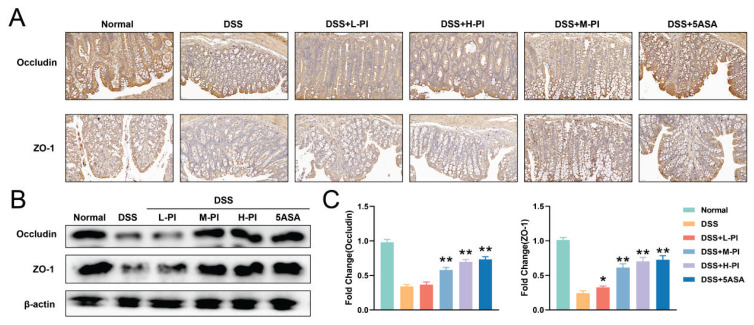
PI enhances the expression of tight junction proteins in UC Mice. **A**) Immunohistochemical staining of ZO-1 and Occludin proteins in the intestines of each group of mice. **B–C**) Western Blot analysis of ZO-1 and Occludin protein levels in the intestines of each group of mice. Data presented as mean±SE (n=10). ** P< 0.01, vs the DSS group.

Tight junction proteins, such as ZO-1 and occludin, play a crucial role in maintaining the integrity of the intestinal mucosal barrier [22]. In this study, we systematically evaluated the expression changes of these tight junction proteins in the colonic tissues of DSS-induced UC mice using immunohistochemical staining and Western blot analysis. The results showed that DSS treatment significantly reduced the expression of ZO-1 and occludin in the colonic tissues of UC model mice, indicating a compromised intestinal mucosal barrier (Fig. 5A).

After treatment with PI, we observed a dose-dependent restoration of the expression levels of these tight junction proteins. Particularly in the high-dose PI treatment group, the expression of ZO-1 and occludin was significantly upregulated, reaching levels close to or comparable with those of the normal control group (Fig. 5B–C). This result clearly demonstrates that PI significantly improves the intestinal mucosal barrier function in UC mice by enhancing the expression of tight junction proteins, thereby increasing the integrity of the intestinal barrier. These findings further support the potential protective role of PI in alleviating UC-related intestinal mucosal barrier damage, especially at higher doses where its effects are most pronounced.

### PI Inhibits UC via the PI3K/AKT Pathway

**Fig. 6:**
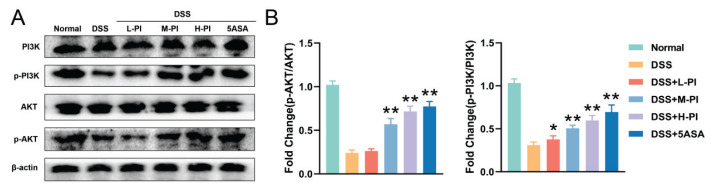
PI Inhibits UC via the PI3K/AKT Pathway. **A–B**) Western Blot analysis of PI3K/AKT-related protein levels in the intestines of each group of mice. Data presented as mean ± SE (n=10). ** P< 0.01, vs the DSS group.

To further elucidate the molecular mechanism by which PI alleviates UC, we focused on the activity changes in the PI3K/AKT signaling pathway based on the results of network pharmacology analysis. Using Western blot analysis, we assessed the expression levels of phosphorylated PI3K (P-PI3K) and phosphorylated AKT (P-AKT) in the colonic tissues of mice following DSS treatment. The results demonstrated that DSS treatment significantly reduced the expression levels of P-PI3K and P-AKT in the colonic tissues of UC model mice, indicating an inhibitory effect of DSS on the PI3K/AKT signaling pathway. However, after treatment with PI, particularly in the high-dose group, the expression levels of P-PI3K and P-AKT were significantly increased, restoring them to levels close to those of the normal group (Fig. 6A–B). This finding suggests that PI effectively activates the PI3K/AKT signaling pathway. Based on these observations, we hypothesize that PI exerts its anti-ulcerative colitis effects by activating the PI3K/AKT signaling pathway. This mechanism may be a key factor in PI’s ability to alleviate UC symptoms, promote the repair of colonic tissues, and restore intestinal barrier function, with more pronounced effects observed at higher doses.

## Discussion

UC is a chronic inflammatory bowel disease that severely impacts patients’ quality of life [1]. Although current treatments alleviate symptoms to some extent, they still have significant limitations and side effects. PI, a natural compound with broad biological activity, has been shown to have anti-inflammatory, anti-tumor, and immunomodulatory effects. However, there has been no research on the use of PI in the treatment of UC until now. This study systematically evaluated the therapeutic effects of PI in a DSS-induced mouse model of UC. The results demonstrated that PI significantly alleviates UC symptoms, improves colon length and structure, enhances intestinal barrier function, inhibits the secretion of inflammatory cytokines, reduces oxidative stress, and exerts its effects through the activation of the PI3K/AKT signaling pathway. These findings provide crucial experimental evidence for the potential application of PI in UC treatment, offering a compelling comparison with existing studies. First, we identified 120 potential targets of PI for UC treatment through network pharmacology analysis and revealed its significant roles in various biological processes and pathways, including inflammatory response and PI3K-Akt signaling, through GO and KEGG analyses. Molecular docking results further indicated that PI has strong binding activity with several inflammation-related targets, such as TNF-α, IL-6, and AKT1, providing a theoretical basis for its role in inflammation regulation. PI significantly alleviated DSS-induced weight loss and Disease Activity Index (DAI) scores in a dose-dependent manner, with effects comparable to those of 5-ASA, highlighting its potential in reducing UC symptoms.

ZO-1 is a multifunctional protein that acts as a “scaffold” in tight junctions, connecting membrane proteins like occludin to the cytoskeleton, thereby helping to maintain the stability and function of tight junctions [23, 24]. Occludin is another key membrane protein that directly participates in the formation and maintenance of tight junctions [25, 26]. It strengthens the barrier function of tight junctions through its interaction with ZO-1. In UC, the impairment of intestinal mucosal barrier function is one of the primary factors contributing to the onset and progression of the disease [27]. Studies have shown that the expression of ZO-1 and occludin is often significantly reduced in UC patients [28]. This reduction leads to the disruption of tight junction structures, increased intestinal permeability, and allows harmful substances, pathogens, and inflammatory factors to penetrate the mucosal barrier more easily, exacerbating inflammation and tissue damage [29]. Therefore, ZO-1 and occludin play crucial roles in UC, with their expression levels directly affecting the integrity of the intestinal mucosal barrier. Our research indicates that PI significantly upregulates the expression of tight junction proteins such as ZO-1 and occludin. This suggests that PI can enhance the function of the mechanical barrier, protect the integrity of the intestinal barrier, and subsequently improve intestinal permeability.

Research has shown that levels of pro-inflammatory cytokines such as TNF-α, IL-1β, and IL-6 are significantly elevated in the serum and colonic tissues of patients with UC [30, 31]. These cytokines activate a series of inflammatory signaling pathways, which continuously trigger and amplify inflammatory responses, leading to damage to intestinal epithelial cells and disruption of the mucosal barrier. For instance, TNF-α not only directly participates in the inflammatory response but also promotes the release of other inflammatory mediators, thereby enhancing the inflammatory cascade and exacerbating the severity of the disease [32]. IL-1β and IL-6 also play critical roles in the pathology of UC; they induce inflammation and affect the integrity of the intestinal barrier, increasing intestinal permeability and allowing harmful substances to penetrate the intestinal wall, leading to further inflammation and tissue damage [33–35]. At the same time, oxidative stress is also a key player in the pathogenesis of UC [36]. Oxidative stress refers to the imbalance between the production of free radicals and the body’s ability to counteract their harmful effects through antioxidants, leading to oxidative damage to cells and tissues. In UC patients, the levels of the oxidative stress marker MDA are significantly elevated, indicating increased lipid peroxidation and damage to cell membranes [37]. This oxidative damage not only directly harms intestinal epithelial cells but also disrupts tight junction proteins, further weakening the function of the mucosal barrier [38]. Additionally, the activity of the antioxidant enzyme SOD is usually significantly reduced in UC patients, indicating impaired antioxidant capacity, which further exacerbates the harmful effects of oxidative stress [39]. There is a mutually reinforcing relationship between oxidative stress and inflammation: inflammation can lead to increased oxidative stress, while oxidative stress can intensify the inflammatory response, creating a vicious cycle that drives disease progression [40]. Therefore, regulating the levels of inflammatory cytokines and oxidative stress is crucial for alleviating UC symptoms and protecting the function of the intestinal barrier. Our study found that PI significantly downregulates the expression of inflammatory cytokines TNF-α, IL-1β, and IL-6 in the intestines and serum of UC mice, while also reducing oxidative stress levels.

In UC, the function of the PI3K/AKT signaling pathway is inhibited [41]. This inhibition can lead to a reduced ability of intestinal epithelial cells to proliferate and survive, thereby compromising the integrity of the intestinal mucosal barrier [42]. Additionally, dysregulation of the PI3K/AKT signaling pathway may result in the overexpression of pro-inflammatory cytokines and the amplification of inflammatory responses, further exacerbating damage to intestinal tissues [43]. Studies have shown that in the DSS-induced UC model, the activity of the PI3K/AKT signaling pathway is significantly decreased, which is closely associated with the disruption of the intestinal barrier and the intensification of the inflammatory response. Research indicates that PI may activate the PI3K/AKT signaling pathway, significantly improving the survival rate of intestinal epithelial cells and enhancing the expression of tight junction proteins, thereby effectively repairing and protecting the intestinal mucosal barrier. This suggests that the PI3K/AKT signaling pathway not only plays a crucial role in the pathogenesis and progression of UC but may also serve as an important therapeutic target for UC treatment.

Although our study provides significant insights into the therapeutic potential of PI in DSS-induced ulcerative colitis, several limitations should be acknowledged. First, this study was conducted in a DSS-induced mouse model, which, while widely used and well-established, may not fully replicate the complexity of UC pathogenesis in humans. Further studies using additional preclinical models, such as spontaneous or genetically induced UC models, are warranted to validate our findings. Second, while we demonstrated that PI exerts its effects through the PI3K/AKT signaling pathway, the precise upstream and downstream molecular mechanisms remain to be elucidated. Future studies involving specific inhibitors or gene knockout approaches are needed to clarify the role of the PI3K/AKT pathway in PI-mediated effects. Third, this study primarily focuses on molecular and histological changes; however, clinical translation requires further pharmacokinetic and toxicity evaluations of PI to confirm its safety and efficacy in humans. Lastly, although we compared the PI-treated groups with DSS and 5-ASA groups, additional comparisons with other potential therapeutic agents could further strengthen the significance of PI as a promising treatment option for UC. These limitations will be addressed in future research to build a more comprehensive understanding of PI’s therapeutic mechanisms and its clinical applicability.

## Conclusion

This study provides new experimental evidence for the potential application of PI in the treatment of UC and reveals that its therapeutic effects may be achieved through the regulation of inflammatory cytokines, oxidative stress, and the PI3K/AKT signaling pathway. These findings lay the foundation for the further development and application of PI, with the aim of offering more effective and safer treatment options for UC patients.
